# Impact of Particle Size on Flour Physicochemical Properties and Cookies Sensory Acceptability Made From *Teff* (*Eragrostis tef* (Zucc.) Trotter)

**DOI:** 10.1002/fsn3.72432

**Published:** 2026-10-07

**Authors:** Adugna Lemu Boka, Getachew Neme Tolesa

**Affiliations:** ^1^ Department of Food Technology and Process Engineering Haramaya Institute of Technology, Haramaya University Dire Dawa Ethiopia; ^2^ Department of Food Science and Postharvest Technology Haramaya Institute of Technology, Haramaya University Dire Dawa Ethiopia; ^3^ Department of Food Technology, School of Applied Sciences, Faculty of Sciences The Papua New Guinea University of Technology Lae Morobe Province Papua New Guinea

**Keywords:** angle of repose, bulk density, color, cookies, particle size, *teff* flour

## Abstract

The physical, rheological, functional, chemical, and other characteristics of flour and end products are crucial to the food processing industries. These characteristics are affected by flour variety and particle size. This study explored the effects of particle size and variety on the physicochemical characteristics of *teff* flour and cookie sensory acceptability. A variety of teff grains (*Bora, Jitu,* and *Filagot*) were ground and sieved to create the three flour fractions (360–260, 260–160, and 160–90 μm). The results showed that samples differed significantly (*p* < 0.05) in the majority of the tested variables. The bulk density, angle of repose, and lightness (l*) of teff flours all significantly increased (*p* < 0.05) as particle size decreased. Redness (a*), yellowness (b*), and chroma (c*) all decreased as flour particle size decreased. Among varieties, there are significant differences (*p* < 0.05) in bulk density, lightness, yellowness, chroma, and hue of *teff* flour, and color, flavor, and overall sensory acceptability of cookies. The overall acceptability scores for the cookies ranged widely, from 4.00 (neither like nor dislike) for Filagot of large particle size flour of 260–360 μm (FP1) to 7.00 (liked very much) for Bora of fine particle size flour (BP3) of 90–160 μm.

## Graphical abstract

This study investigated the effects of *teff* flour varieties and particle sizes on flour physicochemical properties and cookie sensory acceptability. Different teff varieties (*Jitu, Bora*, *and Filagot*) undergo milling and sieving processes to separate the flour into three distinct particle size categories: P1 = 260–360 μm, P2 = 160–260 μm, P3 = 90–160 μm. The resulting flours undergo physicochemical properties analysis to measure parameters such as bulk density, angle of repose, color, and mineral content. After the flour was processed through baking in an oven, a detailed sensory attributes (texture, aroma, flavor, and color) analysis of cookies was performed to determine the overall acceptance and preference for the different varieties and particle sizes combinations.

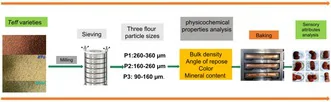

## 1 Introduction

The rheological, physicochemical, and other characteristics of grains and flour are crucial for enhancing the operation and design of food processing equipment used in manufacturing. Identification and analysis of agricultural products' physicochemical characteristics, such as color, bulk density, mineral content, and angle of repose, are crucial. The design and operation of certain machines, for example, grading, cleaning, milling, mixing, drying, and baking, depend on these properties (Wang and Selomulya 2022). It is crucial to comprehend the chemical and physical characteristics of raw and intermediate materials in order to monitor the quality of the finished products (Sun et al. 2023).

The variety and size of flour particles affect the stability of the physical and chemical properties of many food products. According to the researchers, it impacts the particle pack's closeness, which affects the flour bulk density and final product quality, such as bread, cakes, and cookies. Because flour is poorly dispersible, variations in flour particle size can promote stratification, which reduces the quality of the final product (Kaur et al. 2024). Flour variety and particle size can alter pasting properties, water absorption, protein content, and, as a result, baked product quality. Mechanical cereal milling reduces the size of the grain, breaks the bran, germ, and endosperm, and provides distinct flour products (Boz 2019; Qin et al. 2021). These effects result in flour with different particle size distributions and chemical profiles. The wide range in the particle size distribution of flour intended for product formulation appears to be an important area that can have an impact on the quality attributes of the end product (Xiong et al. 2022).

*Teff (Eragrostis teff)* shown in Figure 1, is an ancient tropical cereal grain with several varieties that originated in Ethiopia. It contains all of the amino acids and has more lysine and vitamins than other cereal grains. Because of its unique nutrient content, it offers more nutritional value than other crop cereals or grains and does not require fortification (Legesse et al. 2020).

**FIGURE 1:**
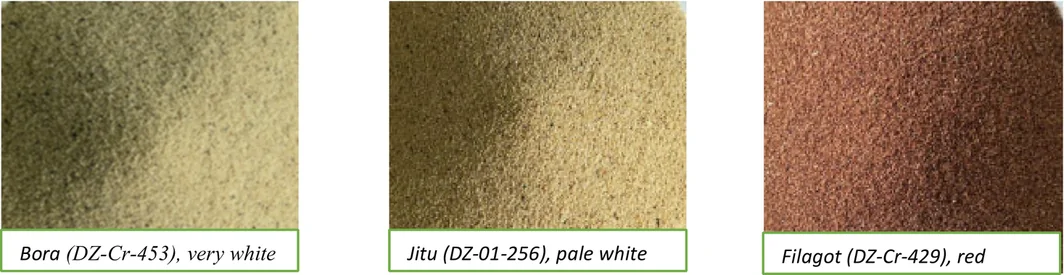
Types of *teff* grain varieties.

Several studies have found that *teff* may be used to produce a variety of gluten‐free foods and drinks, including porridges, pancakes, cakes, weaning foods, cookies, extruded items, and beverages (Woomer and Adedeji 2021). Gluten‐free cookies made with *teff* flour are of superior quality and have antioxidant characteristics (Aguiar et al. 2023). Cookies are made up of three main ingredients: flour, sugar, and fat, and also contain milk, salt, and an aerating agent. Cookies are nutritious, high‐energy snacks produced from dough that is baked in an oven (Raungrusmee et al. 2022).

Despite *teff* having a wonderful nutritional profile, it is mostly consumed in Ethiopia, and there are no detailed global statistics on its production, consumption, and processing (Tadele and Hibistu 2022). Although teff grain agronomy and nutritional profile have been extensively studied, the impact of flour particle size distribution and variety on teff flour's physicochemical properties and sensory acceptability of cookies made from teff has received little study. According to Abebe et al. (2015), the physicochemical and nutritional quality of tef flour is significantly affected by the flour particle size. Villanueva et al. (2022), reported that the sensory analysis of gluten‐free bread from three teff varieties with a combination of maize and bread made with the white variety achieved the highest scores for color, flavor, and texture in the sensory analysis.

The goal of this research was to evaluate the effect of *teff* varieties (*Bora, Jitu*, and *Filagot*) and flour particle sizes (360–260, 260–160, and 160–90 μm) on the physical and chemical properties of *teff* flour and sensory attributes of gluten‐free cookies, to fill a gap in the limit of scientific data or processing parameters, to increase the global use of teff in the future, particularly in areas of food processing industries, as industrial *teff* processing is still under development. It encompasses the milling of clean teff grains followed by mechanical fractionation using standard laboratory test sieves to separate the flour into defined fractions. The findings of this study will be important for the milling and bakery industries.

## 2 Materials and Methods

### 2.1 Experimental Materials

*Bora* (DZ‐Cr‐453), a very white; *Jitu* (DZ‐01‐256), a pale white, and *Filagot* (DZ‐Cr‐429), a red, all (Figure 1) from the Ethiopian Institute of Agricultural Research, Debrezeit Agricultural Research Center.

### 2.2 Experimental Design

The investigation was split into two parts. The first study looked at the effect of variety and flour particle size on physicochemical parameters of *teff* flour, while the second studied the effect of variety and particle size on cookie sensory attributes. The experiment utilized a full factorial design with the two factors (variety and particle size). This consisted of three varieties of *teff* flour (*Bora*, *Filagot*, and *Jitu*) and three fractionations (260–360 μm, 160–260 μm, and 90–160 μm).

### 2.3 Preparation of *Teff* Flour and Cookies

Manual sifting and winnowing are used to remove dust, chaff, and other impurities from *teff* grain. The grain was ground using a local cottage attrition milling method. After sieving using a test sieve screen (JS14S, Zhejiang Tujiang Instrument Co. Ltd., China) connected in tandem with the greatest size on top, three fractions (P1: 260–360 μm, P2: 160–260 μm, and P3: 90–160 μm) were separated, and fractions larger than 360 μm and less than 90 μm were removed. Then, the flour samples were tagged, sealed in polyethylene plastic bags, and kept at room temperature for later work on cookie formulation and laboratory analysis. The cookie ingredients, baking process, baking time, and temperature were done following the methods and formulation with little modification described by Getinet Abebe and Abera (2024).

The cookie was made using the formulation in Table 1. The sugar, salt, and baking powder were combined with a mixer (Kitchen Aid, Philips HR1757 Cucina Blender) for 2 min at 85 rpm. Shortening was then added and blended for two more minutes at 110 rpm. until the mixture became creamy. Then, as indicated in Figure 2, the teff flour was added and blended at 85 rpm for 4 min. To get the desired dough rheology, water was gradually added. The dough was then divided into equal parts and molded. Finally, the cookie dough was baked at 190 °C for 18 min in an oven (DC‐32E Sveba Dahlen AB, Sweden). After cooling, the baked and cooled cookies were packed and tested for sensory acceptability.

**TABLE 1:** Cookies baking ingredients formulation.

Ingredients	Quantity (g)
*Teff* flour	100
Sugar	45
Shortening	45
Salt	1
Baking soda	2
Water	20 mL

**FIGURE 2:**
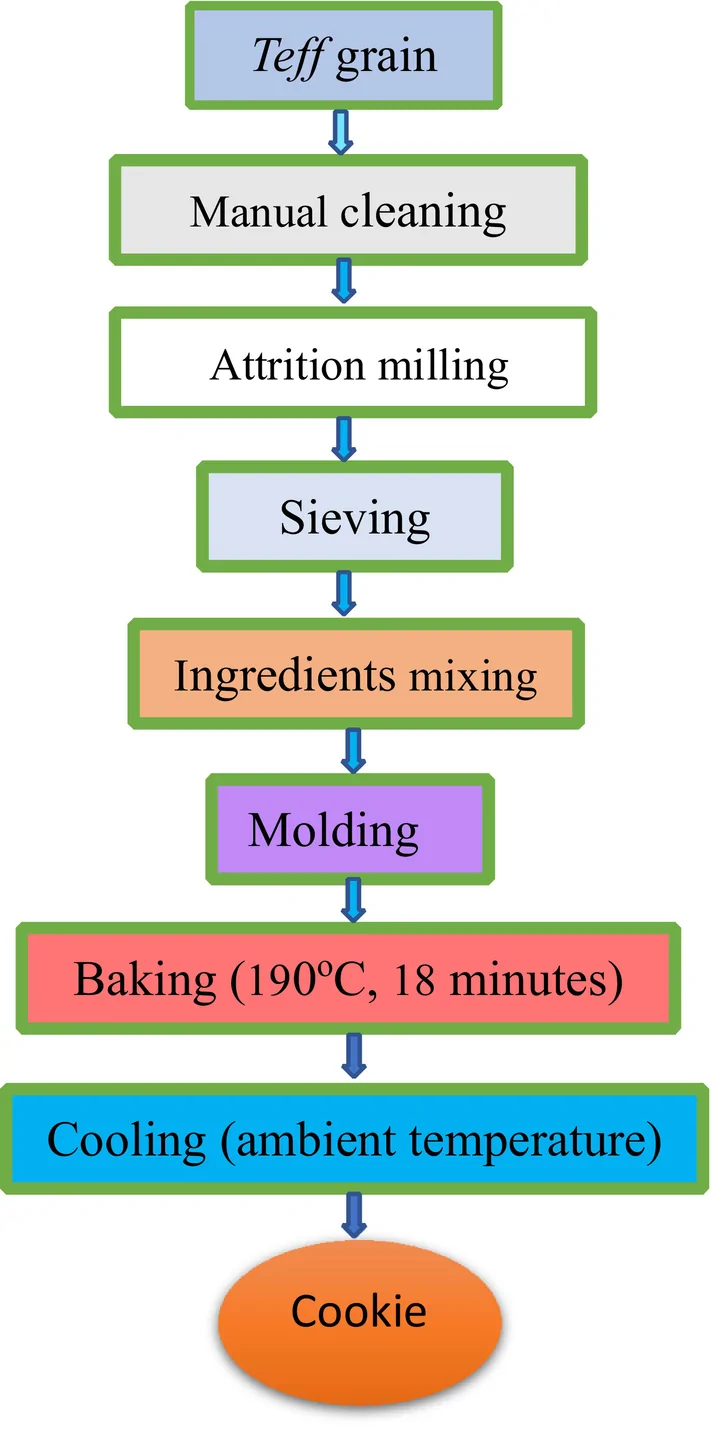
Preparation of flour and cookies from *teff* flour.

### 2.4 Determination of Physicochemical Properties of *Teff* Flour

#### 2.4.1 Moisture Content of *Teff* Flour

The moisture content of the *teff* flour was determined using the AOAC (2016) standard method.

#### 2.4.2 Bulk Density of *Teff* Flour

The bulk density of *teff* flour was determined using the Yu et al. (2024) method. A 100 mL measuring cylinder was filled with flour until the capacity reached 100 mL. The mass of the measuring cylinder was calculated by subtracting the weight of the flour. The bulk density (*ρ*) was calculated using the following formula:

(1) $$\text{Bulk density of flour}\;\rho \;\left(g/\mathrm{mL}\right)=\frac{\text{weight of sample}\;\left(g\right)}{\text{volume of sample}\left(\mathrm{mL}\right)}$$

#### 2.4.3 Angle of Repose of *Teff* Flour

A cylinder was used in the funnel method to calculate the grain and flour's angle of repose (Al‐Hashemi and Al‐Amoudi 2018). The cylinder containing the *teff* flour was set on a level surface. A conical heap with a natural slope was created as the cylinder slid and was progressively raised off the ground. The angle of repose (^o^) was determined by measuring the base diameter and the height of the heap above the ground.

(2) $$\text{The angle of repose}\;\left(Ф\right)=\tan^{-1}\;\left(\frac{2h}{d}\right)$$

where Ф = angle of repose (°), h = height of the pile (mm), and d = diameter of the pile (mm).

#### 2.4.4 Color of *Teff* Flour

Based on the tristimulus value, the color of the *teff* flour was measured using the Hunter *L, a, b* scale color space method. The values for lightness (*L*), redness (+Ve *a*), yellowness (+Ve *b*), and the magnitude of overall color difference were calculated using (ARS00006, Hunter Lab Aeros Spectrophotometer, USA) as L*, a*, and b* values (Hryhorenko et al. 2023).

(3) $$\mathrm{Hue}\;\text{angle}=\tan^{-1}\;\left(\frac{b^{*}}{a^{*}}\right)$$

(4) $$\text{Chroma}=\sqrt{{\left(a^{*}\right)}^{2}+{\left(b^{*}\right)}^{2}}$$

#### 2.4.5 Determination of Mineral Content of *Teff* Flour

Some selected minerals, such as Calcium, Iron, and Zink were determined by American official methods of analysis using an atomic absorption spectrophotometer (210VGP, Buck Scientific, USA) (AOAC 2016).

### 2.5 Evaluation of the Sensory Acceptability of Cookies

Sensory evaluation of cookies was conducted within 24 h after baking. Thirty panels who attended a Food Sensory evaluation and Product Development course evaluated cookies for color, texture, crispness, taste, flavor, and overall sensory acceptability. These were selected from students and teachers in the Food Technology and Process Engineering, and Food Science and Post‐harvest Technology Departments. All panels were adults, including 17 men and 13 females. Selected product samples from each were provided to the panelists in a random order on a white plate, with no indication about the product type. Before and after examining each sample, the panelists were requested to rinse their mouths with water. It was assessed using a seven‐point hedonic scale that represents 7 (Like very much), 6 (Like moderately), 5 (Like slightly), 4 (Neither like nor dislike), 3 (Dislike slightly), 2 (Dislike moderately), and 1 (Dislike very much).

### 2.6 Statistical Analysis

Utilizing a two‐way Analysis of Variance (ANOVA) and a factorial test, the statistical data were assessed using the Statistical Analysis System (SAS) for Windows (SAS Institute Inc., Cary, C, USA). *p* < 0.05 was established as the cutoff point for a significant test. To compare the means, Fisher's Least Significant Difference (LSD) analysis was used. The results, along with the mean and standard deviation (mean ± standard deviation), were shown in triplicate.

## 3 Results and Discussion

### 3.1 The Effects of Varieties and Particle Size Distribution on the Physical Properties of *Teff* Flour

The physical properties of *teff* flour are displayed in Table 2 below. Moisture content describes how much water is in a product. It has an impact on the flour's physical characteristics. Among varieties, there are significant differences (*p* < 0.05) in the moisture content of *teff* flour. The values for *Bora*, *Filagot*, and *Jitu* were 10.01%, 9.05%, and 8.05%, respectively. Variations in chemical makeup, storage conditions, and genetics could all be contributing factors to the moisture content variability. Getinet Abebe and Abera (2024) reported a *teff* moisture content of 8.95%, which was in close agreement with the observed results. Comparably, Abera et al. (2021) discovered that different rice varieties had different levels of moisture. For processing and storage, this information is useful because moisture content directly impacts the shelf life of flour and grain. Longer storage times are possible with lower moisture content. However, it is usually not appropriate for long‐term storage when the moisture content exceeds 10%. The moisture content was not significantly impacted (*p* > 0.05) by the flour's particle size.

**TABLE 2:** Effects of varieties and particle size on physical properties of *teff* flour.

Factors	Physical properties of *Teff* Flour		
Varieties	Moisture content (%) db	Bulk density (g/ml)	Angle of repose (^o^)
*Bora*	10.01 ± 0.07^a^	0.74 ± 0.07^c^	39.73 ± 1.95^ab^
*Filagot*	9.05 ± 0.07^b^	0.83 ± 0.07^a^	39.93 ± 1.73^a^
*Jitu*	8.05 ± 0.07^c^	0.77 ± 0.04^b^	39.28 ± 2.22^b^
Particle size (μm)			
260–360	9.05 ± 0.81^a^	0.72 ± 0.04^c^	37.40 ± 0.84^c^
160–260	9.01 ± 0.84^a^	0.77 ± 0.03^b^	39.74 ± 0.66^b^
90–160	9.05 ± 0.89^a^	0.86 ± 0.05^a^	41.80 ± 0.23^a^
LSD	0.06	0.01	0.57
CV	0.71	1.78	1.45

*Note:* All values are means of triplicate ± standard deviation. Values with different superscripts within the same column are significantly (*p* ≤ 0.05) different. a,b,c superscript letters show significantly different within the same column (*p* < 0.05).

Bulk density determines both product porosity and particle expansion. Varieties of *teff* flour showed significant (*p* < 0.05) variations in bulk density. The sample of the *Filagot* variety had the highest bulk density (0.83 g/mL), while the *Bora* variety had the lowest (0.74 g/mL), as shown in table 2. According to Náthia‐Neves et al. (2023), the variation can be caused by the content of the flour. Similarly, the bulk density of flour was significantly (*p* < 0.05) affected by particle size. As the flour's particle size decreased from 360 to 90 μm, its bulk density increased from 0.72 to 0.86 g/mL. As the particle sizes decrease, the pore space decreases, and the bulk density increases because the smaller particles occupy the spaces left by the bigger ones. A high bulk density indicates low porosity and significant compaction of the flour. The findings align with previous figures by Lin et al. (2022) that the bulk density of sugar beet powder increased from 0.45 to 0.62 g/mL as flour particle size decreased. The size and spatial arrangement of the particles that make up flour can affect its bulk density (Kaiser et al. 2019).

There was a significant (*p* < 0.05) difference in the flour's particle size in the angle of repose of *teff* flour (Table 2). Compared to other particle size flour samples, the 90–160 μm flour classification exhibited a greater angle of repose (41.80°). The findings show that when the size of the flour particles decreased from 260 to 360 μm to 90–160 μm, the angle of repose of the flour increased from 37.40 to 41.80^o^. A cohesive or poor flow resulted from more surface area being accessible for the interaction of cohesive forces or strengths because the particle surface area per unit mass increased with decreasing particle size. For this reason, tougher flowability was linked to smaller particle sizes.

Smaller particles hinder flow by increasing the surface area accessible for the interaction of cohesive surface forces and friction (Jan et al. 2018). Barretto et al. (2023) discovered that the flowability of chickpea flour decreased and its angle of repose increased when the flour particles decreased. When designing a hopper, the angle of repose is crucial. The hopper's inclination must be greater than the flour's angle of repose in order to maintain continuous flow to prevent clogging.

### 3.2 Effects of Varieties and Particle Size on the Color of *Teff* Flour

Table 3 shows the observed color values for different varieties and particle sizes of *teff* flour. L*, a*, and b* varied from 64.74 to 70.29, 2.91 to 6.35, and 15.68 to 18.61, respectively. The *Bora* variety had the highest recorded lightness, while the *Filagot* type had the lowest. The grains' color was the cause of the discrepancy in the outcomes. *Filagot* was red, while *Bora* was quite white. The red grain color and the pigment in the cover dispersing throughout the flour during milling were the reasons for the lower lightness L* value score of the *Filagot* flour. *Filagot*'s high a* value of 6.35, in contrast to the L* value, was significantly (*p* < 0.05) different from *Bora*'s 2.91 and *Jitu*'s 2.93. Because both *Bora* and *Jitu* were white, their values did not differ significantly. Flour yellowness (b* value) was high for *Filagot* and low for *Jitu*. The results are in agreement with the reported values by Alemneh et al. (2022) for different varieties of *teff* flour.

**TABLE 3:** Effects of flour varieties and particle size distribution on *teff* flour color.

Factors	Color Scales				
Varieties	L*	a*	b*	c*	h*
*Bora*	70.29 ± 0.80^a^	2.91 ± 0.51^b^	17.39 ± 0.53^b^	17.63 ± 0.60^b^	80.27 ± 1.26^a^
*Filagot*	64.74 ± 0.52^c^	6.35 ± 0.50^a^	18.61 ± 0.67^a^	19.66 ± 0.77^a^	74.47 ± 1.01^c^
*Jitu*	68.48 ± 1.13^b^	2.93 ± 0.49^b^	15.68 ± 0.73^c^	15.95 ± 0.81^c^	79.23 ± 1.01^b^
LSD	0.21	0.19	0.02	0.06	0.34
Particle size (μm)					
260–360	66.88 ± 2.26^c^	4.62 ± 1.67^a^	17.94 ± 1.22^a^	18.57 ± 1.59^a^	76.77 ± 2.56^c^
160–260	67.90 ± 2.49^b^	4.04 ± 1.69^b^	17.27 ± 1.30^b^	17.79 ± 1.61^b^	77.99 ± 2.80^b^
90–160	68.73 ± 2.63^a^	3.53 ± 1.79^c^	16.46 ± 1.30^c^	16.89 ± 1.62^c^	79.21 ± 2.71^a^
LSD	0.20	0.19	0.01	0.06	0.34
CV	0.30	4.73	0.08	0.36	0.44

*Note:* All values are means of triplicate ± standard deviation. Values with different superscripts within the same column are significantly (*p* ≤ 0.05) different L*: lightness; a*: redness/greenness; b*: yellowness/blueness; c*: chroma; h*: hue. a,b,c superscript letters show significantly different within the same column (*p* < 0.05).

The distance perpendicular to the coordinate's lightness is known as the chromaticity coordinate or chroma. Varieties had significant effects on the chroma (c*) value (*p* < 0.05), as shown in Table 3. The physical properties of grains, like color, play a significant role in the variations in flour color among genotypes. *Filagot* had the greatest reported chroma (19.66), while *Jitu* had the lowest (15.95). *Bora*, *Filagot*, and *Jitu* had Hue angle values of 80, 74, and 79 for whole *teff* flour, respectively. The findings align with already reported whole oat flour values of 18.8 chromas and 75.4 colors (Silventoinen‐Veijalainen et al. 2024). It was anticipated that the various *teff* flour samples would differ in color since the composition is influenced by breed, variety, location, and growing conditions.

Particle size distribution also had significant effects (*p* < 0.05) on the color of fractionated *teff* flour (Table 3). One important aspect influencing materials utilized for light‐emitting applications is particle size. Light is either absorbed less or scattered (reflected) more when the particle size is smaller. The lightness (L*) values ranged from 66.88 to 68.73, with samples measuring 90–160 μm (P3) having the greatest value and samples measuring 260–360 μm (P1) having the lowest. This indicates that particle size can affect the red‐green and yellow‐blue values of the flour's surface. The a* values ranged from 3.53 to 4.62, and the b* values ranged from 16.46 to 17.94.

The l* values increased as the *teff* flour particle size decreased, whereas the a* and b* values decreased significantly (*p* < 0.05). This may have to do with the smaller particles' fine pores and great homogeneity, which reduce light absorption and increase light reflection, making the flour lighter and less red and yellow. Fernando and Manthey (2021) found that changes in sample particle sizes brought on by various mill settings may be the cause of variation in the lightness and yellowness of black bean flour.

Additionally, significant (*p* < 0.05) differences in hue and chroma values were noted among flour particle sizes. Hue and flour particle size are negatively correlated. This might be the case because, in contrast to small flour, which has low saturation and higher light or bright values, large particles generate saturated color with high chroma and low hue values (Sebastião et al. 2023). Because it is lighter and less reddish than other flours, the 90–160 μm flour is the most desirable flour type in terms of color characteristics.

### 3.3 Effects of Flour Varieties and Particle Size Distribution on Selected Mineral Contents of *Teff* Flour

Minerals and other micronutrients are essential for evaluating the nutritional value of *teff*. They serve as cofactors in several physiological and metabolic processes, making them vital parts of the human diet (Alemneh et al. 2022). *Teff* flour variations had a significant (*p* < 0.05) impact on the mineral contents, as shown in Table 4. Calcium, iron, and zinc concentrations of 132, 14.31, and 4.14 mg/100 g were significantly (*p* < 0.05) greater in the *Filagot* variety. Similarly, the highest average iron content of 14.31 mg/100 g was recorded for the *Filagot* flour sample.

**TABLE 4:** Effects of varieties and particle size on mineral composition of *teff* flour.

Factors	Mineral contents of *Teff* Flour		
Varieties	Calcium(mg/100 g)	Iron(mg/100 g)	Zinc(mg/100 g)
*Bora*	115.04 ± 0.03^b^	7.26 ± 0.01^c^	3.20 ± 0.01^b^
*Filagot*	132.10 ± 0.03^a^	14.31 ± 0.00^a^	4.14 ± 0.00^a^
*Jitu*	114.02 ± 0.01^c^	8.27 ± 0.01^b^	3.18 ± 0.01^c^
Particle size (μm)			
260–360	120.40 ± 8.80^a^	9.94 ± 3.30^a^	3.51 ± 0.47^a^
160–260	120.39 ± 8.80^a^	9.95 ± 3.30^a^	3.50 ± 0.47^a^
90–160	120.38 ± 8.78^a^	9.93 ± 3.29^a^	3.51 ± 0.48^a^
LSD	0.02	0.01	0.006
CV	0.02	0.12	0.18

*Note:* All values are means of triplicate ± standard deviation. Values with different superscripts within the same column are significantly (*p* ≤ 0.05) different. a,b,c superscript letters show significantly different within the same column (*p* < 0.05).

Climate and variety are two of the many factors that affect the mineral content of *teff* (Yilmaz and Arslan 2018). The iron red and white flour levels are consistent with the previously published findings of Kerie et al. (2024), who discovered that red *teff* flour had 24.72 mg/100 g and white *teff* flour had 8.81 mg/100 g. The findings were consistent with those of Culetu et al. (2021), who found that the calcium, iron, and zinc concentrations of *teff* flour were 166.70, 8.67, and 4.00 mg/100 g, respectively. Particle size has an insignificant (*p* > 0.05) impact on *teff* flour's mineral content.

### 3.4 Effects of Varieties and Particle Size Distribution on Sensory Acceptability of Cookies

Food quality was examined using human‐perceivable sensory characteristics, such as color, appearance, flavor, taste, crispness, and texture, which also served as standards when choosing foods (Li et al. 2025). Seven hedonic scales were used to assess the acceptability of cookies' sensory attributes. Table 5 displays the panelists' assessments of the sensory evaluation results, including color, appearance, sharpness, taste, flavor, and overall acceptability.

**TABLE 5:** Effects of varieties and particle size on sensory acceptability of cookies.

Factor variety	Sensory attributes of cookies					
	Color	Texture	Crispness	Taste	Flavor	Overall acceptability
*Bora*	5.92 ± 0.70^a^	5.85 ± 0.64^a^	6.17 ± 0.46^a^	5.78 ± 0.71^a^	5.85 ± 0.67^a^	6.35 ± 0.65^a^
*Filagot*	4.35 ± 0.81^c^	5.23 ± 0.58^b^	6.05 ± 0.87^a^	5.75 ± 0.45^a^	4.98 ± 0.66^c^	4.43 ± 0.49^c^
*Jitu*	5.42 ± 0.49^b^	5.32 ± 0.53^b^	6.03 ± 0.48^a^	5.38 ± 0.49^b^	5.27 ± 0.49^b^	5.37 ± 0.48^b^
Particle size (μm)						
260–360	4.66 ± 0.76^c^	5.17 ± 0.51^c^	5.74 ± 0.50^c^	5.54 ± 0.52^b^	5.32 ± 0.63^b^	4.90 ± 0.75^c^
160–260	5.18 ± 0.87^b^	5.34 ± 0.56^b^	6.01 ± 0.55^b^	5.58 ± 0.49^b^	5.16 ± 0.58^b^	5.26 ± 0.95^b^
90–160	5.84 ± 0.80^a^	5.88 ± 0.64^a^	6.51 ± 0.60^a^	5.80 ± 0.70^a^	5.63 ± 0.82^a^	6.00 ± 0.82^a^
LSD	0.13	0.14	0.16	0.14	0.16	0.08
CV	9.04	9.11	9.00	9.01	10.16	5.51

*Note:* All values are means of triplicate ± standard deviation. Values with different superscripts within the same column are significantly (*p* ≤ 0.05) different. a,b,c superscript letters show significantly different within the same column (*p* < 0.05).

#### 3.4.1 Color

Figure 3 shows cookies made from three *teff* varieties in different particle sizes or three flour fractions for each. The *Bora* variety of *teff* cookies earned the best score (5.92) statistically (*p* < 0.05), while the *Filagot* had the lowest score (4.35). The color acceptability scores of the cookies changed significantly (*p* < 0.05) as a result of the particle size distribution. Samples with particle sizes between 90 and 160 μm had the highest mean value of 5.84, while samples with particle sizes between 260 and 360 μm had the lowest score of 4.66. Color is an essential component of any product's visual quality and has a significant impact on consumer decisions and quality evaluation. Color differences can be utilized as a crucial quality metric to verify changes in the structure or composition of a material or product. The *Filagot* sample's cookies had the lowest color acceptance scores, which could be attributed to the skin's natural red tint, which lowers consumer preference. Color variations can be utilized as a crucial criterion to verify changes in a material's or product's structure or makeup (Ageh et al. 2025).

**FIGURE 3:**
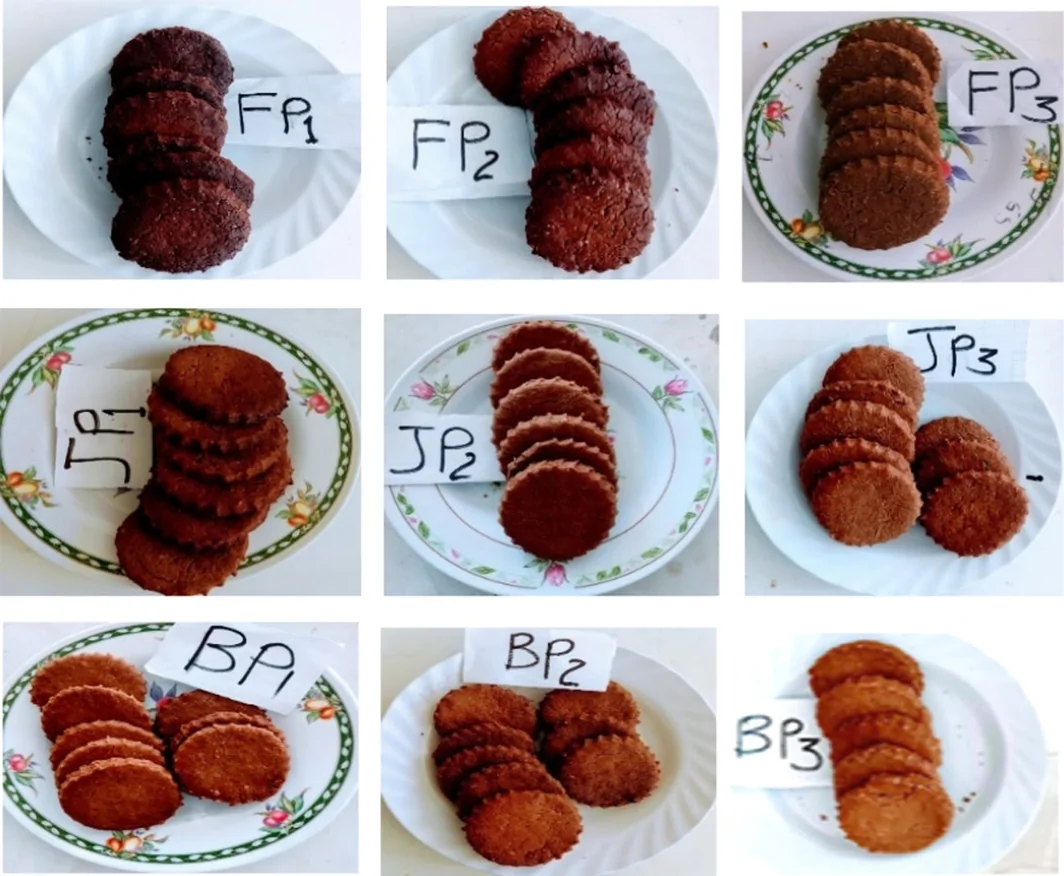
Cookies made from different varieties of *teff* flour. F‐Filagot, J‐Jitu and B‐Bora varieties; P1 = 260–360 μm, P2 = 160–260 μm, P3 = 90–160 μm.

#### 3.4.2 Texture

An essential organoleptic characteristic of cookies that influences their quality and consumer acceptability is their texture. Variety and flour particle size distribution had a significant impact. (*p* < 0.05) on the acceptability scores for cookie texture. The highest rank was 5.85 for cookies made of the *Bora* variety and 5.88 for those produced from the finest particles (90–160 μm), while the lowest rank was 5.23 for cookies made from the *Filagot* variety and 5.17 for those formed from large flour particles (250–360 μm). Similarly, Attenborough et al. (2023) found that flour particle size distribution affected the texture of corn extrudates.

#### 3.4.3 Crispness

Table 5 displays the recorded mean crispness results, which ranged from 6.03 to 6.17 and did not differ significantly (*p* > 0.05) from one another due to varieties. The crispness of the cookie was significantly (*p* < 0.05) impacted by the particle size, with the greatest score of 6.51 for cookies made with flours with particle sizes between 90 and 160 μm and the lowest score of 5.74 for cookies made with flours with particle sizes between 260 and 360 μm. The flour particle size and crispness had a negative relationship. This relation may be due to the increase in surface area, leading to greater starch damage, which results in a thinner, more brittle, and crispier texture as particle size decreases. The crispiness is defined as the force and noise with which a product breaks rather than deforms when eaten with the teeth. During chewing, force, compression energy, and point of rupture all increased as particle size increased; therefore, the samples became harder and less crispy. According to Sharifi et al. (2021), there is a significant negative correlation between flour particle size and snack crispness.

#### 3.4.4 Taste and Flavor

Taste is the perception of sensory cells in taste buds. The brain detects various tastes and flavors when food molecules activate these sensory cells. There is a statistically significant difference (*p* < 0.5) between the greatest and lowest taste sensory acceptance scores for both factors. Varieties of flour had a significant (*p* < 0.05) impact on the cookies' flavor. The *Bora* variety had the greatest score of 5.85, while the *Filagot* variety received the lowest sensory acceptability value of 4.98. There were significant (*p* < 0.05) differences in flavor acceptability scores of the cookies due to the particle size of the flour. The 90–160 μm sample scored higher acceptability than the two‐particle size flours. This could be because when particle size reduces, flavoring compounds interact with other substances or ingredients to produce a stronger flavor.

#### 3.4.5 Overall Acceptability

Variety caused significant (*p* < 0.05) differences in the overall acceptance scores of cookies (Table 5). A cookie made using *Bora teff* flour scored as high as 6.35, while the *Filagot* variety scored as low as 4.43. Similarly, there were significant (*p* < 0.05) differences in the overall acceptability of cookies as a result of flour particle size.

Those with flour particles between 90 and 160 μm had the highest value (6.00), while those with particles between 260 and 360 μm had the lowest value of 4.90. Across all sensory assessments, the cookies (Figure 3) manufactured with the *Bora* flour variety and those with flour fractions between 90 and 160 μm received the highest score. In terms of texture, flavor, crispness, and general acceptability, these cookies scored the highest values. The findings generally indicate that consumers preferred the cookies' higher level of crispness and whiter color. Kong et al. (2026) reported a comparable finding of wheat cookies' sensory acceptability for cookies made from small particle size flour.

## 4 Conclusion

The variety and particle size distribution of *teff* flour have a significant (*p* < 0.05) impact on *teff* flour and baked cookies properties. Among these properties, the bulk density, angle of repose, and lightness (L*), redness (a*), yellowness (b*), and chroma (c*) of *teff* flour and sensory attributes of cookies vary considerably. These variations can have an impact on surface area, composition, color, and starch quality, which in turn affects the structure of the flour's constituents and, ultimately, the quality of cookies. The flour angle of repose showed that as the flour's particle size reduced, the flow pattern shifted from free to fair flowing, showing a negative correlation. Among the *teff* varieties, *Filagot* has a significant mineral abundance. As the flour's particle size decreased, it became lighter. The finest flour particle size (90–160 μm) had distinct physical properties when compared to flours with larger particle sizes (260–360 μm). Varieties and flour particle size had a significant effect (*p* < 0.05) on human‐detectable sensory characteristics of cookies, including color, appearance, flavor, taste, crispness, and texture. As the particle size of *teff* flour decreased, all characteristics improved. The cookies' overall acceptance scores ranged from 4.00 (neither like nor dislike) for *Filagot*'s large particle size flour of 260–360 μm (FP1) to 7.00 (liked very much) for *Bora*'s fine particle size flour of 90–160 μm (BP3).

## Author Contributions

**Adugna Lemu Boka:** conceptualization, writing – original draft, methodology, software, writing – review and editing, formal analysis. **Getachew Neme Tolesa:** methodology, investigation, supervision, formal analysis.

## Funding

The work was supported by the Federal Democratic Republic of Ethiopia, the Ministry of Education, and Haramaya University, Ethiopia.

## Conflicts of Interest

The authors declare no conflicts of interest.

## Data Availability

The data supporting the findings of the study are accessible from the corresponding author upon reasonable request.
